# Raw transcriptomics data to gene specific SSRs: a validated free bioinformatics workflow for biologists

**DOI:** 10.1038/s41598-020-75270-8

**Published:** 2020-10-26

**Authors:** D. N. U. Naranpanawa, C. H. W. M. R. B. Chandrasekara, P. C. G. Bandaranayake, A. U. Bandaranayake

**Affiliations:** 1 Agricultural Biotechnology Centre, Faculty of Agriculture, University of Peradeniya, Peradeniya, 20400 Sri Lanka; 2 Postgraduate Institute of Science, University of Peradeniya, Peradeniya, 20400 Sri Lanka; 3 Department of Computer Engineering, Faculty of Engineering, University of Peradeniya, Peradeniya, 20400 Sri Lanka

**Keywords:** Computational platforms and environments, Data acquisition, Data integration, Data mining, Data processing, Genome informatics, Sequence annotation, Software

## Abstract

Recent advances in next-generation sequencing technologies have paved the path for a considerable amount of sequencing data at a relatively low cost. This has revolutionized the genomics and transcriptomics studies. However, different challenges are now created in handling such data with available bioinformatics platforms both in assembly and downstream analysis performed in order to infer correct biological meaning. Though there are a handful of commercial software and tools for some of the procedures, cost of such tools has made them prohibitive for most research laboratories. While individual open-source or free software tools are available for most of the bioinformatics applications, those components usually operate standalone and are not combined for a user-friendly workflow. Therefore, beginners in bioinformatics might find analysis procedures starting from raw sequence data too complicated and time-consuming with the associated learning-curve. Here, we outline a procedure for de novo transcriptome assembly and Simple Sequence Repeats (SSR) primer design solely based on tools that are available online for free use. For validation of the developed workflow, we used Illumina HiSeq reads of different tissue samples of *Santalum album* (sandalwood), generated from a previous transcriptomics project. A portion of the designed primers were tested in the lab with relevant samples and all of them successfully amplified the targeted regions. The presented bioinformatics workflow can accurately assemble quality transcriptomes and develop gene specific SSRs. Beginner biologists and researchers in bioinformatics can easily utilize this workflow for research purposes.

## Background

During the past decade, DNA and RNA sequencing technologies have made tremendous progress, in terms of throughput, speed and reduction of sequencing cost^1^. Similarly, access to genomes and transcriptomes have greatly benefited animal and plant biology research^2–7^. Sequencing technologies have evolved faster than one can expect. Second or Next Generation (NGS), and third generation sequencing technologies which are currently in use^8–10^ (Table 1) boast a vast improvement from the first-generation sequencing technologies, especially with regard to throughput/cost ratio and speed. Traditional Sanger sequencing, which was widely used for almost three decades since its publication in 1977, could only achieve limited or very low throughput^11^. The human genome project employed Sanger sequencing methods, and required over 10 years and nearly US$3 billion for completion^12^. In contrast, the Illumina HiSeq system can now sequence over 45 human genomes for US$1000 each in a single day^13^. Illumina is one of the NGS technologies where its HiSeq X instrument can achieve up to 900 Gb of total throughput at cost per Gb of a merely US$7^14^. While Illumina is considered the most popular NGS technology for short-read sequencing^14, 15^, the second generation also includes other technologies such as Ion torrent and SOLiD by Life Technologies, where the throughput can reach up to 15 Gb^16^ and 155 Gb, respectively^17^. Third generation sequencing methods such as SMRT sequencing by Pacific Biosciences (PacBio)^18^ has the potential to produce around 50 Gb of data per SMRT cell with reads as long as 190 kb^19^. Nanopore sequencing by Oxford Nanopore can generate very long read-lengths^20,21^, and their PromethION platform promises a yield up to 15 Tb of data in 2 days^22^. Further, it would only cost US$900 for a Nanopore platform to decipher around a billion DNA bases^23,24^.

**Table 1:** Maximum throughputs recorded for sequencing platforms.

Sequencing instrument	Throughput
**Illumina**	
NextSeq	120 Gb^25^
HiSeq X	900 Gb^14^
Ion torrent	15 Gb^16^
SOLiD	155 Gb^17^
PacBio	50 Gb^19^
PromethION	15 Tb^22^

Due to these drastic advances, the amount of raw sequence reads produced by the sequencers are huge, and the high coverage adds up a massive amount of overlapping fragments of DNA/RNA, especially in large genomes^26^. Because the volume of data to be handled is very high, assembling the short reads back to construct the complete genome or transcriptome becomes challenging, requiring high computational power and execution time. This leads to a significant bottleneck in computational biology and bioinformatics^27^.

Assembly of raw sequence data follows either of two approaches: (1) reference based^28,29^ and (2) de novo assembly^30–35^. Reference based assembly, also called comparative assembly, is the process of recreating the genome or transcriptome using prior knowledge. In this method, a previously assembled genome of a closely related organism is used as a template to map or align the sequenced reads in question. Every read is placed at its most likely position against the reference assembly. The resulting assembly could be similar to the reference but not completely identical as there could be regions that are significantly different^36^. Therefore, comparative assembly is mostly used in genome re-sequencing projects and is considered a computationally easy task^37^.

Assembling sequence reads with no prior knowledge of the transcriptome or without a reference genome is called de novo assembly. While de novo assembly provides the opportunity to assemble any novel organism, the process presents many challenges^38^ including segmental duplicates, sequence repeats, missing or fragmented genes, and the massive amount of raw reads to be handled. Applying de novo assembly methods for plant genomes gives rise to even more limitations due to the size and complexity of plant genomes compared to animal genomes^39^. Furthermore, de novo assembly is mathematically proven to be difficult, given that it belongs to a family of problems with NP-hard complexity for which no efficient solution is known yet^40^. Nonetheless, de novo assembly is widely used over comparative assembly since many complex organisms are yet to be sequenced and closely related reference genomes are not always available^41,42^.

While there are many applications and uses of assembled transcriptome of an organism, the identification of molecular markers^43,44^ plays an important role in breeding programs that amplifies plant characteristics such as resistance and yield^45–47^. Of the different types of molecular markers, microsatellites^48^—also known as simple sequence repeats (SSR)—have been utilized most extensively. Transcriptome based SSRs have now replaced genome based SSRs because it is more effective and less expensive, and a number of such microsatellite markers have been published^49–52^.

Even though sequencing technologies have advanced rapidly in a short span of time, methods and software used for assembly and analyses of sequence data^37, 53^ have not seen the same degree of improvement. While most of these tools are still being revised for better algorithmic approaches and efficiency^54–56^, the knowledge gap in bioinformatics has not allowed the rate of improvement to increase. One of the main reasons for this limitation lies in the fact that these assembly and annotation software are mostly commercial and very expensive^57^. However, free and open-source software play a major role in bridging this gap, not only by allowing anyone to test and experiment with their data, but also by allowing them to make changes and suggest improvements for the said tools. Even so, there is a lack of identified complete workflows built using such free software. This is again a setback for novice biologists as the workflow up to analysis of raw sequence data might contain several different procedures, and each individual tool might take up a considerable amount of time to decipher its workings.

Currently there are many free pipelines and frameworks available for transcriptome assembly using RNA-seq data. Galaxy^58^ is a popular web-based online platform to build scientific workflows using preconfigured tools within. Considering it is a shared resource on public servers, the disk space quota and the number of concurrent analysis jobs an individual can have at a time are limited^59^. To address privacy and space issues Galaxy offers commercial clouds, which might prove to be expensive^60^. Even though Academic clouds are also provided, configuration of the platform is complex. A complementary web-based tool to Galaxy is Taverna^61^, which allows the integrating of third-party web-service tools to a Galaxy workflow. In addition to being fairly network intensive, Taverna also has limitations on the size of data it can handle. It is inherently targeted at a more expert audience and might require specific knowledge such as handling servlet containers and Java servlets. Hence, setting up, configuration and usage of both Galaxy and Taverna is not easy for a beginner with little to no computer science background. Open-source, cloud-based workflows are also available for RNA-seq analysis such as Arvados^62^, Agave^63^ and RAP^64^. However, one of the main disadvantages of these is that they impose a lot of constraints on the user as to the number of analyses that can be performed, and the size of raw data that can be uploaded to the cloud. In addition, they have retention policies where the data uploaded by the user will only be maintained at the cloud server for a limited amount of time after analyses have been performed. Cloud-infrastructure also demands high-speed internet connections for file transfer. Therefore, if such infrastructure is not available for the user, they might be discouraged from using such cloud computing environments. Bpipe^65^ is a command line programming language that has been designed for defining and executing bioinformatics pipelines including transcriptome assembly. However, the user has to learn a completely new set of syntax and semantics specific for the Bpipe platform in order to use it efficiently. Nextflow^66^ is another command line application that can be used for various RNA-seq analyses. However, the transcriptome assembly process of Nextflow is reference-based and cannot be used for de novo assembly. TransFlow^67^ is a framework providing five independent modules that can be combined to build different transcriptome assembly workflows. Conversely, it requires the user to configure and specify parameters such as *kmer* lengths for assemblers for optimizations, which might be difficult for a beginner to decide at first.

In essence, most of the currently available frameworks for de novo transcriptome assembly are too complicated for a beginner biologist to start analyzing RNA-seq data immediately. Further, the outcome of most of these pipelines is only the transcriptome assembly, and does not extend into any further analysis. Conversely, there are a lot of tools available for post-processing and analyzing the assemblies with applications such as SSR marker designing and annotation^68,69^. However, majority of these do not provide any pipeline for de novo assembly from raw reads and expect the user to make available the assembled genome or transcriptome.

Here we present a complete workflow of free software that can be executed in a Linux environment. All the tools that we have used were developed by various other research groups and scientists, and are freely available online to download and execute (Supplementary File 01). Some of the software are even open-source, which allows users to suggest improvements and fix bugs, inherently improving the performance of the tools in return. Our workflow spans from acquiring sequenced reads, through quality control and assembly of data, up to assembly quality assessment and SSR primer design. For the current study, transcriptomic data was downloaded from a published study on *Santalum album* (sandalwood)^70^. We generated SSRs targeting few important oil biosynthetic genes with the objective of identifying makers for future breeding efforts. A batch of designed primers were validated with laboratory experiments.

## Methods

### Data acquisition

The majority of the sequencing data generated through sequencing projects are deposited at the Sequence Read Archive (SRA)^71^ maintained by the United States National Institutes of Health National Center for Biotechnology Information (NIH/NCBI). The NCBI hosts a multitude of resources for bioinformatics and provides access to over 35 sequence databases^72^ including GenBank and PubMed. GenBank also coordinates with repositories maintained by European Molecular Biology Laboratory (EMBL) and DNA Data Bank of Japan (DDBJ). Between these primary databases, SRA contains more than 10,000 terabases of raw sequence data^73^ as of 2018. NCBI also provides the SRA Toolkit software^74^ freely. This can also be used to perform various sequence read file manipulation operations including downloading raw reads from NCBI and converting data file formats to suit various processing requirements.

For the current work, we used previously published transcriptomic data (BioProject PRJNA297453) of *S. album* (sandalwood) generated from four oil-producing sandalwood trees for a study of exploring the biosynthetic enzymes of key components of sandalwood fragrance^70^, and the group isolated RNA from the tissues of three development stages of the trees; Sapwood (SW), Transition Zone (TZ), and Heartwood (HW). The *S. album* data were paired-end Illumina reads with a total of 117.98 Giga Bases in size.

The dataset (Supplementary file 02) was directly accessed through the FTP directory hosted by NCBI^75^, and *prefetch* and *fastq-dump* commands of the SRA Toolkit were used to download each read file (Supplementary file 03). If the required dataset is small, Linux *wget* command can be used to download them directly. In contrast, the Toolkit can be used to download large datasets that span several libraries and experiments. After downloading, the SRA Toolkit was used to split the paired-end *sra* files into its respective forward and reverse read files in *fastq* format.

### Quality control of data

After acquiring data, it is essential to identify basic statistics and the quality of the reads before proceeding into assembly and downstream analyses since low quality reads will result in low quality assemblies. We used the Linux command-line version of the FastQC package^76^ to identify and evaluate the read quality by processing the *fastq* files (Supplementary file 04). FastQC is an open-source tool compatible with all main sequencing platforms. It allows the observation of read quality across all raw reads of a sample including diagnostics such as GC content distribution, average base quality per score, and adapter content. Reports generated by FastQC are supported by visualizing plots and accompanied by warnings about uncertain results.

After evaluating the quality based on the reports provided by FastQC, the reads that did not meet the defined standards were filtered using FASTQ quality filter in FASTX-toolkit^77^ (Supplementary file 04). FASTX can remove reads or nucleotides that are below a certain threshold specified by the user based on the insights gained from FastQC. If FastQC reports indicate high adapter content, scripts included in Trimmomatic^78^ can be used to trim such adapter sequences introduced by NGS instruments when sequencing (Supplementary file 05).

In some specialized cases such as assembling RNA-seq data, additional steps can be taken to clean up the reads. RNA-seq data could include ribosomal RNA (rRNA), which could cause errors in downstream analyses if not removed at early stages. To clean such rRNA that might be present in the dataset, we used SortMeRNA^79^ (Supplementary file 06). After performing read filtering, FastQC reports were generated again to assess if previously reported warnings have been resolved and if the read quality has improved.

### De novo* transcriptome assembly*

We chose Trinity de novo assembler^80^ for the assembly of the *S. album* RNA-seq dataset. Trinity is considered to provide high quality assemblies^81^ and consists of three software modules: Inchworm, Chrysalis and Butterfly. These stages work in an automated flow resulting in a complete transcriptome assembly.

For paired-end reads, Trinity takes two fastq files as its input; a forward end read file and a reverse end read file. Since multiple RNA-seq files from different libraries were available in our dataset, all forward end reads and all reverse end reads were concatenated into two separate fastq files by using simple Linux commands (Supplementary file 08). Attention was given in setting parameters such as maximum memory to be used by Trinity (–max_memory), number of CPUs to use (–CPU), and normalizing reads (–no_normalize_reads). Memory and CPU usage depends on the hardware platform that Trinity has been installed on. Normalizing is usually required for large datasets with deep sequencing as redundant data could be present. We set default values for all transcriptome assembly parameters and set the dataset to be normalized with in silico normalization^82^ provided by the Trinity pipeline (Supplementary file 07, Supplementary file 08). The assembly was run on a server with 32 cores and 128 GB of Random Access Memory.

Trinity assembles the raw reads into ‘contigs’. A contig is the smallest assembly component and it represents sets of overlapping DNA that can be summed to form a contiguous region of DNA. For further analysis, these contigs are required to be clustered into larger sequences – called 'unigenes' within the Trinity environment. This process is internally carried out by Trinity without user intervention. If further processing is required, Trinity provides scripts to avoid redundant transcripts and filter the 'longest isoform genes' as well.

### Assessing and validating assembly quality

As de novo assembly is performed without no prior information available, quality assessment is a critical step. Using a low-quality assembly would not only misrepresent the results of any proceeding experiments, but it would also lower the reliability, credibility and repeatability of any related analyses.

Initial quality assessment was done with a Perl script provided in Trinity to retrieve basic statistics about the assembly. This script was run on the *fasta* file containing the transcriptome assembly, which generated the values for total length of assembly, number of contigs assembled, GC content, and N50 statistics of contigs and unigenes.

Further quality assessments were done with several other software (Supplementary file 09). The Bowtie2 program^83^ was used to align the input sequence reads back to the transcriptome assembly obtained with Trinity. From the list of quality values provided by Bowtie2 after alignment, the percentage of raw reads mapped back to the transcriptome was observed primarily to assess the assembly quality. In addition, BUSCO assessment tool^84^ was used to perform an evolutionary measure of genome completeness by searching the assembly against a reference database. For the current study, ‘eukaryota_odb9′ reference database was downloaded from the BUSCO website which was the database closest to the species of interest. The statistics from the validation include the percentage of complete orthologs (single copy and duplicates), fragmented orthologs, and missing orthologs as well. The value generated for the number of complete orthologs was given priority for assessing the assembly quality. Furthermore, TransRate program^85^ was also used to produce a number of program-specific quality metrics to further validate the assembly. Attention was given to the percentage of good mappings and assembly score to assess completeness.

### Designing SSR primers for identified genes

For this study, we selected eight predicted oil biosynthetic genes of *S. album*^86^ and two control genes (rbcL and TUB1), and the coding sequences of the preferred genes were downloaded from NCBI in *fasta* format. Then, using Linux command-line ncbi-blast + tool (ftp://ftp.ncbi.nlm.nih.gov/blast/executables/blast + /), a blast database was created for the local transcriptome assembly generated by Trinity, which acts as a subject for following queries. Each gene sequence was then individually queried against the local database via the *blastn* command of ncbi-blast + and the significant alignments were directed into a separate text file. Sequence IDs of most significant alignments for each gene were subsequently filtered from the text file, and the list of IDs were used to extract the aligned sequences from the transcriptome assembly in *fasta* format.

Some of the selected genes showed duplicate alignments to the transcriptome. To ensure that we choose unique sequences for primer design, we used the Basic Local Alignment Search Tool (BLAST) web interface of the NCBI, which provides the facility to query against a published genome (https://blast.ncbi.nlm.nih.gov/Blast.cgi). The preferred genome could be mentioned in the search bar under ‘BLAST Genomes’ by the organism name, scientific name, or tax id. Here, we used the *S. album* (taxid: 35,974) genome published under BioProject PRJNA411901. Sequences that were earlier extracted from the transcriptome assembly, were individually queried against the genome to determine the best sequence for primer design. Candidates with a unique alignment and lowest E-value were selected.

The BatchPrimer3 (UC Davis Server)^87^ and OligoAnalyzer^88^ tools were used for detecting SSR markers and designing primers. On BatchPrimer3, default parameters were used with ‘SSR screening and primers' as the primer type, and the chosen candidate sequence was the input. The minimum number of SSR pattern repeats for di, tri, tetra, penta, and hexa nucleotide SSR types were specified. From the resulting list, primers were evaluated considering GC content, primer melting temperature (Tm) and product length. The selected forward primer and reverse primer were separately analyzed by using the web tool OligoAnalyzer by observing the characteristics such as hairpins, Tm and GC percentage.

The forward and reverse primers were again queried separately against the selected *S. album* genome to assure unique and specific amplification, and such primers were selected.

### Laboratory validation of designed SSRs

We conducted wet lab experiments to assess the accuracy of the de novo transcriptome assembly as well as the SSR primer design process. DNA was extracted from bark samples following hexadecyltrimethyl ammonium bromide (CTAB) method^89^. PCR was carried out in a 25 µL reaction volume containing lx PCR buffer, 1.5 mM MgCl2, 200 µM dNTP (Promega, Cat No: U1515), 0.2 µM of each primer (Integrated DNA technologies, Singapore), 100 ng of DNA, 0.8 µM spermidine and 1 Unit Go Taq Flexi DNA polymerase (Promega, Cat No: M8295). The PCR cycle consisted of 94 °C of initial denaturation for 3 min, followed by 35 cycles of 94 °C for 1 min, annealing temperature ranging from 55 °C-58 °C for 30 s (depending on primer) and 72 °C for 30 s and final extension at 72 °C for 5 min. Amplified products were separated by electrophoresis (5 V cm-1) on 2% agarose gels and Safegreen (abm G108-G) stained gels were visualized and photographed using ChemiDoc XRS^+^ system with Image Lab Software (version 6.0.1.34), where excitation source was UV trans Illumination and emission filter was a standard filter. Further the products were separated using 8% polyacrylamide gel electrophoresis for higher resolution. Sizes of the PCR products were estimated with a 100-bp DNA molecular weight marker (promega G2101). In addition to that, a similar PCR experiment was conducted with three *S. album* accessions to check the polymorphism of the SSR marker set and applicability in future breeding work.

**Figure 1:**
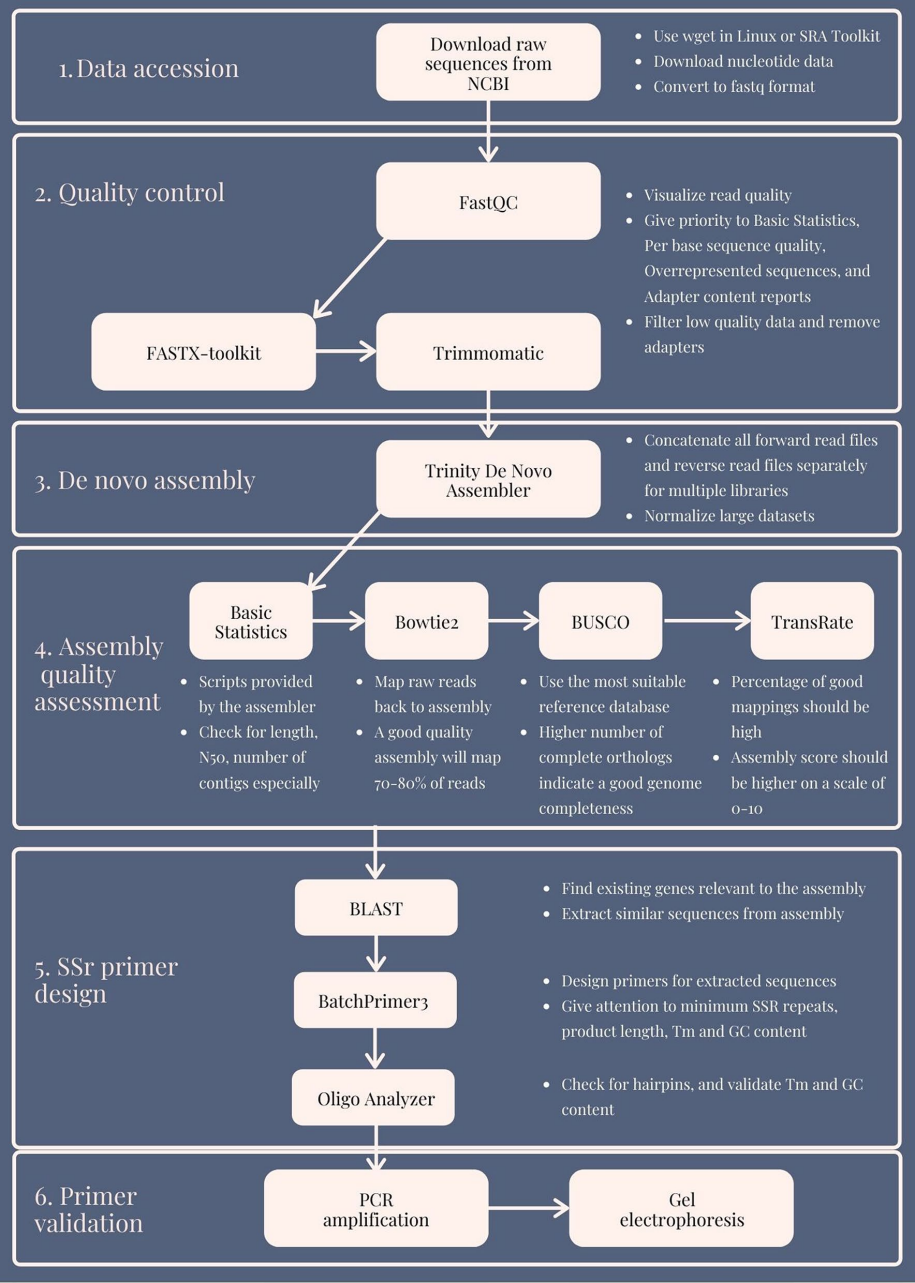
A summarized computational workflow for de novo transcriptome assembly, and quality assessment and assembly validation.

A summarized view of the complete assembly workflow is presented in Fig. 1.

## Results

### Data acquisition and quality control of raw reads

NCBI data is in SRA format by default when downloading. If the raw data is paired-end, the *sra* files need to be converted and split into its respective forward and reverse read files in *fastq* format for use of processing tools. If the data is single-end the conversion can be direct without splitting.

On the other hand, the above step is not applicable if the researcher has generated own sequencing data. Depending on facilities produced by the service provider, sequenced data may directly be downloaded in *fastq* format from a storage hosting service such as a cloud, or from the sequencing instrument.

**Figure 2:**
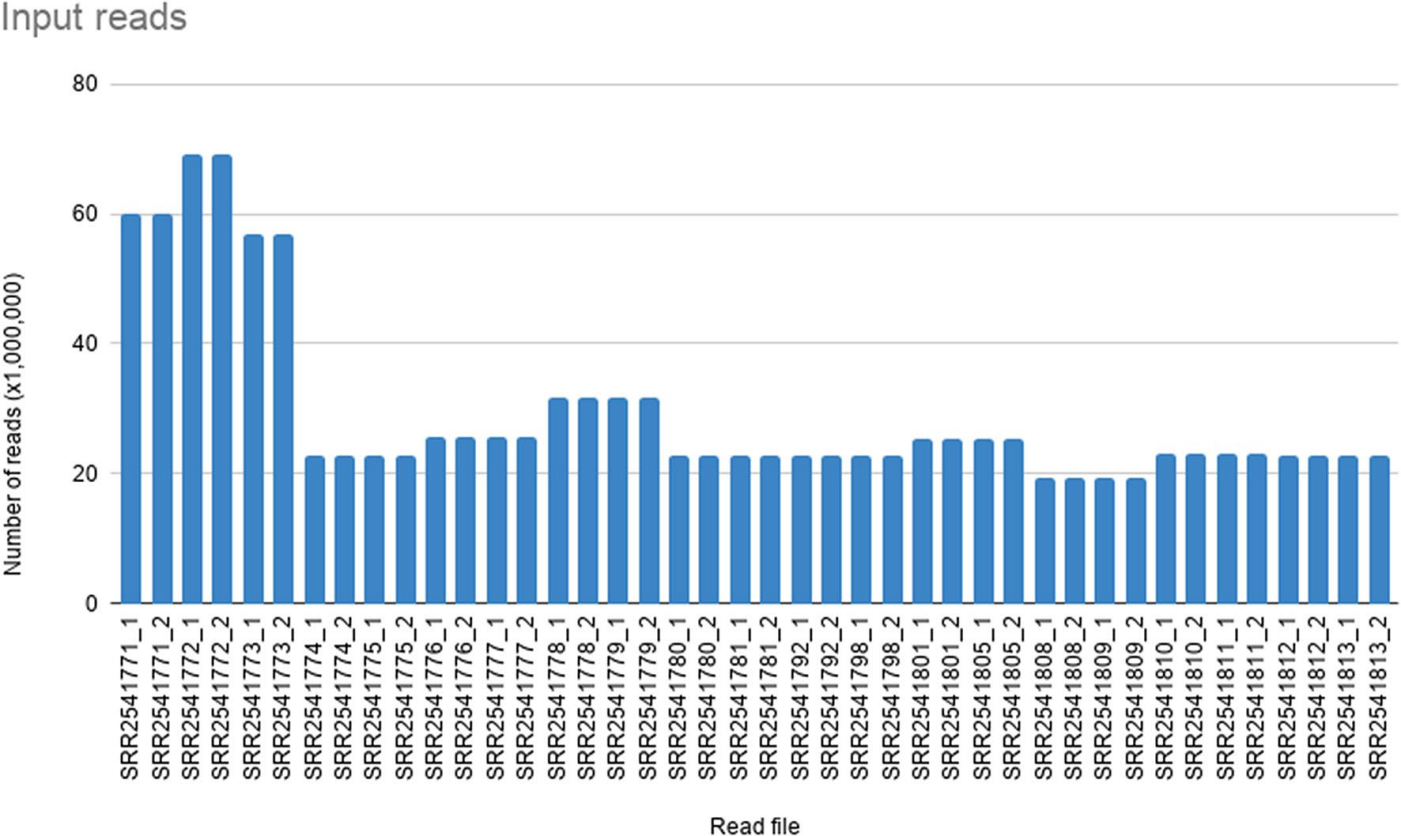
Variation in the number of raw reads in input RNA. Each accession file has a forward and reverse dataset indicated by suffixes _1 and _2 respectively.

**Figure 3:**
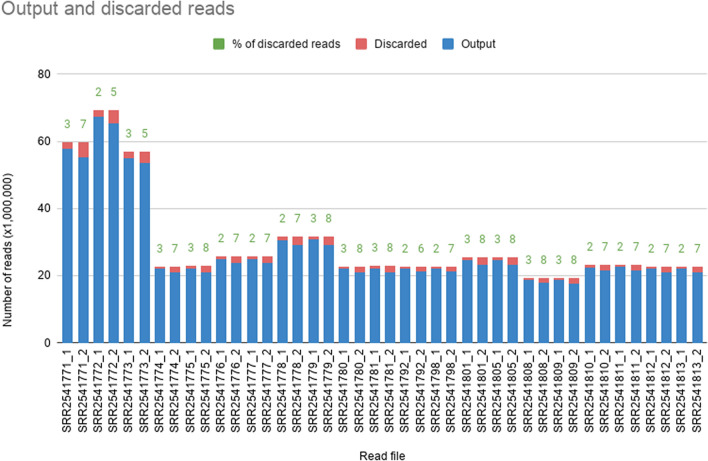
Variation in the number of raw reads in the output after filtering the original data with FASTX toolkit, and the number of filtered reads. Filtered reads as a percentage of input reads are also indicated. Each accession file has a forward and reverse dataset indicated by suffixes _1 and _2 respectively.

The RNA-seq dataset of *S. album* downloaded from NCBI had a complete volume of 117.98 Gbases and 53.42 GB for 21 SRA accessions. Number of raw reads in forward and reverse read files of each SRA accession are given in Fig. 2. When the raw reads were assessed with FastQC with default parameters, generated reports indicated a certain level of low-quality reads based on the ‘Per base Sequence Quality’, ‘Overrepresented sequences’, and ‘Adapter Sequences’ metrics. The number of raw reads after filtering low quality reads with FASTX-toolkit are indicated in Fig. 3. Filtered and trimmed read files indicated only good quality reads were remaining upon rechecking with FastQC.

### De novo* transcriptome assembly*

Using Trinity, a total of 628,438,851 bases were assembled into 771,200 contigs. Default values for all parameters were provided for the execution of the assembly command. The generated contigs were further clustered within Trinity assembly process into 604,666 unigenes with a mean length of 561 bp and N50 value of 659 (Table 2). Total assembly execution time for Trinity was 167,335 s with Chrysalis stage taking a major portion of that time (160,011 s).

**Table 2:** Summary statistics of sequence data and De novo assembly.

Description	Statistics
**Sequence reads**	
Raw reads	117.98 Gb
**Assembly**	
GC content (%)	39.13
**Contigs**	771,200
Number of bases	628,438,851
Mean length (bp)	814.88
N50	1405
**Unigenes**	604,666
Number of bases	339,069,641
Mean length (bp)	560.76
N50	659

### Quality assessment of the transcriptome assembly

While basic quality metrics do provide an idea about the contiguity of the assembly, the applicability of them might change according to the type of assembly and amount of data.

The basic statistics generated by Trinity indicated that a total of 628,438,851 bases were assembled as contigs with an N50 of 1405. From the contig bases, only 339,069,641 bases were further clustered into unigenes with an N50 of 659 (Table 2).

The length of the assembly and the number of contigs generated provide a straightforward insight into the success of the assembly. Lower the number of contigs, better the assembly – but all the contigs as a group should cover a majority of estimated assembly size for accuracy. Previous studies or flow-cytometry analysis may help to estimate the assembly size beforehand. For an example, previous studies suggest that a chloroplast genome assembly would normally be between 120 kb (kilobases) to 170 kb in length^90–92^. Hence, if basic statistics of a chloroplast genome exceeds or falls below that general range, it is best to be revised again. The same concept applies for transcriptome assemblies as well.

The GC content of a sequence represents the percentage of nucleotide bases that are either guanine or cytosine on a DNA or RNA molecule. DNA sequences also include adenine and thymine bases, while RNA has uracil instead of thymine. A gene-rich genome can be roughly identified with the GC content, as it is indicative of many protein-coding genes. Low percentages of GC might indicate a large amount of non-coding DNA in the genome^93^. Knowing the GC content of an assembly or a sequence is also important for downstream experiments such as polymerase chain reactions (PCR)^94^ and primer design^95^ as their annealing temperatures might have to be determined. A higher GC percentage indicates a higher melting temperature.

N50 is a weighted median statistic provided for assessing the contiguity of an assembly fragmented by contigs of different sizes. The value is defined as the minimum contig length required to cover half of the genome or transcriptome^96^. That is, 50% of the sequence length is contained in contigs equal to or larger than the N50 value in length. Considering the total number of bases assembled (~ 628 Mb), the N50 of current analysis does not indicate a good quality assembly as it is considerably low. However, due to lowly expressed isoforms, N50 metric could behave in a biased manner^97^. In addition, even though N50 might convey a sense of scale and contiguity of the assembly, it does not correlate with the accuracy or the coverage of the assembly as demonstrated by recent large-scale assembly competitions^98–100^. In some cases, a large N50 value might be produced artificially due to large contigs that might have been misassembled^100^. Contigs might need to be corrected for erroneous concatenations and N50 measured again for a more accurate indication of contiguity. All the same, N50 by itself cannot be guaranteed as a good measure for transcriptome assembly quality and does not necessarily indicate accurate contig orientation^98,101^.

Bowtie2 execution on the transcriptome assembly indicated 83.45% of proper pairs out of the RNA-seq fragments aligned to the transcriptome (Table 3). Only 10.2% of the fragments were recorded as improper pairs.

**Table 3:** Summary of statistics of Bowtie validation.

Read type	Count	%
Proper pairs	145,103,817	83.45
Improper pairs	17,730,957	10.2
Right only	5,553,993	3.19
Left only	5,495,262	3.16
Total aligned rnaseq fragments		173,884,029

Bowtie2 program^83^ provides statistics as to how many cleaned reads actually represent the assembly. Assembly quality is high if at least 70–80% of sequence reads were mapped back to the assembly by Bowtie2^102^. The current assembly quality can be considered high since more than 80% of the reads indicated to be proper pairs. Given that the assembly was generated from many short reads as well as reads with average quality, this alignment is justifiable for the quality of the transcriptome.

BUSCO is a tool to assess completeness of genome assembly, gene set and transcriptome. It is based on the concept of single-copy orthologs that should be highly conserved among the closely related species. The BUSCO assessment reported that out of the 303 BUSCO groups searched using a global reference, 91.7% of the *S. album* transcriptome were complete orthologs, while only 1.4% were missing (Table 4).

**Table 4:** Summary of statistics of BUSCO validation.

BUSCO type	Count	%
Complete	278	91.7
Complete and single-copy	78	25.7
Complete and duplicated	200	66
Fragmented	21	6.9
Missing	4	1.4
Total BUSCO groups searched	303	

The high percentage of complete single-copy orthologs generated by the BUSCO assessment tool^84^ suggests a high quality and near-complete assembly since more than 90% of complete orthologs were present. The higher number of duplicated orthologs out of the complete orthologs indicates that multiple copies of full-length orthologs are found in the assembly. Since this is a transcriptome assembly, where multiple sequences are reconstructed at varying levels of abundance, this metric can be considered normal.

The TransRate assembly score was also considerably low at only 0.0105 and out of all RNA-seq fragments, only a 0.16% of fragments can be considered as good mappings (Table 5).

**Table 5:** Summary of statistics of TransRate validation.

Read mapping metrics	Value
Fragments	181,844,966
Fragments mapped	31,616,682
Fragments mapped %	0.17
Good mappings	28,474,573
Good mappings %	0.16
Transrate assembly score	0.0105
Transrate optimal score	0.0618
Good contigs	318,291
Good contigs %	0.41

Therefore, in contrast to validations performed by both Bowtie2 and BUSCO, TransRate indicated poor assembly quality. The percentage of good mappings and assembly score indicates the level of assembly completeness^85^. The assembly score ranges from 0 to 10 according to the specifications of TransRate, and a higher assembly score would indicate a higher quality transcriptome assembly. Previous studies indicate that there is a threshold of 0.22 in the TransRate score which was achieved by only 50% of the 155 published de novo transcriptomes in NCBI^85^. The resulting score for current assembly is below this threshold. However, as in the case of the N50 metric, TransRate metrics could also be biased against a large number of lowly expressed transcripts, leading into a lower score^97^. More sequence data or information might be required to correct or improve these TransRate scores.

### Validation of designed SSRs with PCR amplification

SSR primers that were designed for selected sandalwood genes and controls are presented in Table 6.

**Table 6:** SSR motifs and primers designed for 10 gene sequences.

	GenBank Accession	Definition	SSR Motif	Primer Sequence 5′ to 3'		Product size
1	KC842188.1	cytochrome P450 reductase (CPR2)	(GTGC)2	Forward	ATGCCCTCTGTTTAAGCTACT	151
				Reverse	GAACAGAGTCAATCAGATCGT	
2	KT160233.1	SaCPR722 cytochrome P450 reductase	(GGAAA)2	Forward	CAGCGAGGTTTTATAAATGG	146
				Reverse	CTCAGAAAGAATGTCATCCAC	
3	KT160234.1	*S. album* isolate SaCPR3442 cytochrome P450 reductase	(ACAG)2	Forward	CAGCGAGGTTTTATAAATGG	146
				Reverse	CTCAGAAAGAATGTCATCCAC	
4	KT160235.1	*S. album* isolate SaCPR7351 cytochrome P450 reductase	(TCGGC)2	Forward	AGTGGACTACGAGGATGAGTT	152
				Reverse	CATGAACATCACGAAACCTAC	
5	KT160236.1	*S. album* isolate SaCytB5-4631 cytochrome b5	(TTTCTT)2	Forward	CTCTCTCTCGATTCTGTTGTG	162
				Reverse	TATTTACTGGGCACACCTATG	
6	KT160237.1	*S. album* isolate SaCytB5-6548 cytochrome b5	(ATGG)2	Forward	ATAACGCTTCAGGAATAGGAC	137
				Reverse	CCCTCTGCTGTTAAAGATGAT	
7	KT160238.1	*S. album* isolate SaCytB5-3125 cytochrome b5	(TAGTA)2	Forward	TTCTCAATCCTTAGACCCACT	135
				Reverse	GACGGTTCAGATGCAAGTAT	
8	KT160239.1	*S. album* isolate SaCytB5-5956 cytochrome b5	(CACAA)2	Forward	AAGTTCCGTTCTCTCTGAATC	143
				Reverse	GAGGTTAGATTGTAAACCTTCC	
9	rBcL	ribulose-1,5-bisphosphate carboxylase/oxygenase large subunit [*Arabidopsis thaliana* (thale cress)]	(AGTTCA)2	Forward	GTCCGATGGGATAGACTAAAA	159
				Reverse	GTTCAACCAACCCATTTTC	
10	TUB1	tubulin beta-1 chain [*Arabidopsis thaliana* (thale cress)]	(TCAA)2	Forward	GGTTGAGATCACCAACTGTAA	146
				Reverse	CCTTATGAAACATGCTTTGG	

Using command-line ncbi-blast + tool on the Linux system seemed to be highly efficient than using the NCBI website for blastn queries. The NCBI web tool depends on the available network bandwidth and is congested frequently. In addition, the process would take too long, and saving results for later use is arduous. Instead, by using the Linux application for blast executions and simple Linux commands, files can easily be manipulated into providing succinct, exact outputs.

The *S. album* genome published in NCBI (BioProject PRJNA411901, Master accession NXEK01000000) was used to observe unique alignments of candidate sequences. When designing primers for these sequences on BatchPrimer3, minimum repeats for di, tri, tetra, penta, and hexa SSRs were set as 2, 2, 3, 3, and 3 respectively. From the results, eight pairs of forward and reverse primer sequences were tested unique against the *S. album* genome on NCBI (Table 6).

**Figure 4:**
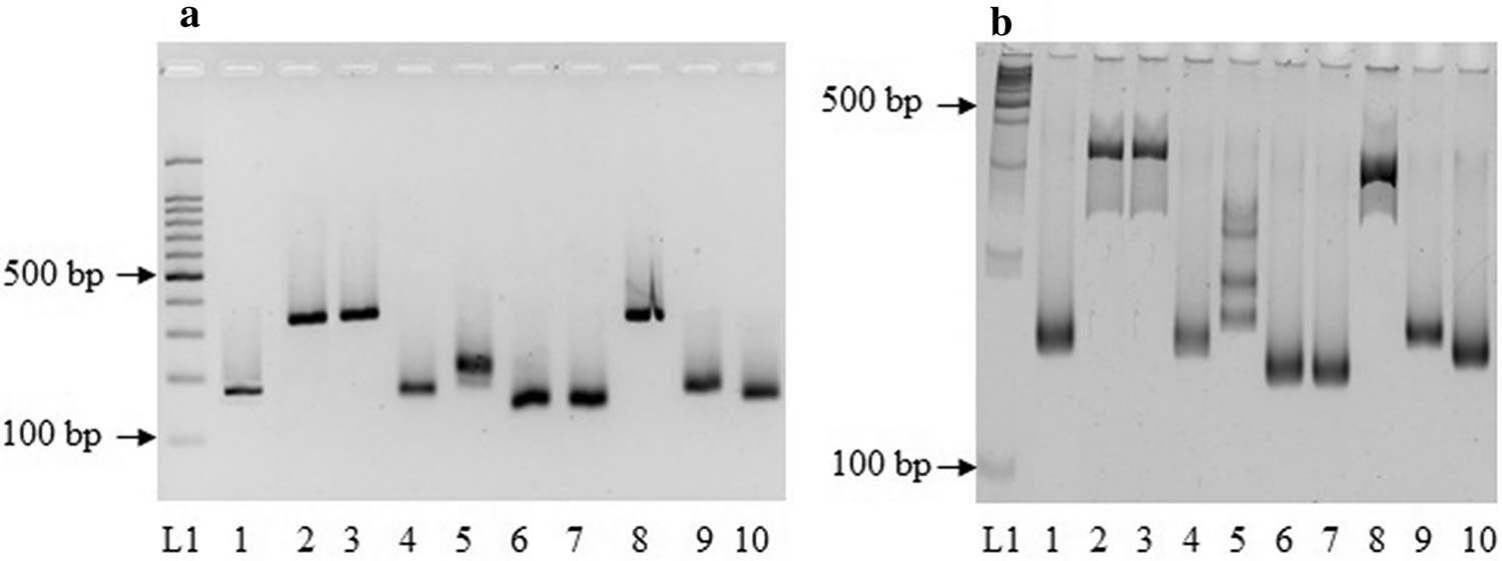
Polymerase chain reaction amplification of Simple Sequence Repeat markers and two housekeeping genes of *S. album*. (A): Agarose gel electrophoresis, (B): Polyacrylamide gel electrophoresis (PAGE). L1:100 bp molecular weight marker (promega G2101), 1:KC842188.1, 2:KT160233.1, 3:KT160234.1, 4:KT160235.1, 5:KT160236.1, 6: KT160237.1, 7:KT160238.1, 8:KT160239.1, 9:rBcL, 10 :TUB1. Full length gel image is presented in the Supplementary file 10.

All the eight SSR primer pairs amplified expected fragments from genomic DNA of selected *S. album* accession including control primers, rbcL and TUB1. While KC842188, KT160235, KT160237, and KT160239 amplified at 55 ℃, others amplified at 59 ℃. All the SSR markers except KT160236 appeared as single alleles in both the agarose and polyacrylamide gels, suggesting these novel markers possess a specific amplification in *S. album* (Fig. 4). However actual product sizes were greater than the expected sizes for KT160233, KT160234 and KT160239.

**Figure 5:**
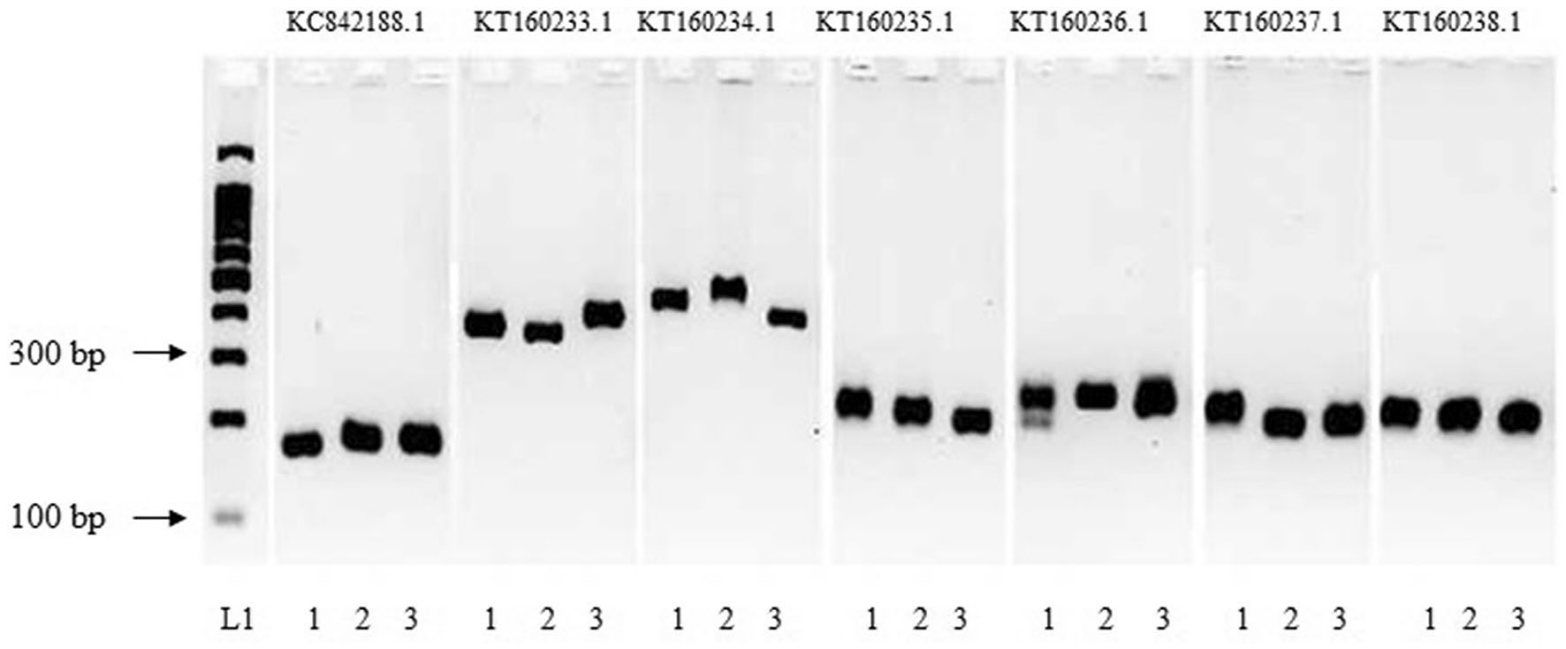
Agarose gel electrophoresis of Simple Sequence Repeats amplified products of three Sri Lankan *S. album* accessions. L:100 bp molecular weight marker (promega G2101), 1–3: *S. album* accessions. Full length gel image is presented in the Supplementary file 11.

We further tested the polymorphism of selected primers with the objective of optimizing them for future breeding efforts. Of them, KT160233, KT160234, KT160235 and KT160237 resulted length polymorphism among three selected accessions (Fig. 5). This suggested high polymorphism among *S. album* accessions, as well as the ability to detect such variations with primers designed.

## Discussion

We presented a complete workflow of free software from acquiring sequenced reads, through quality control and assembly of data, up to assembly quality assessment and SSR primer design. All the tools used are freely available online to download and execute while some of the software are open-source.

Quality control of raw reads is essential because sequencing instruments could introduce impurities during the sequencing process which would propagate into the final data output. In addition, sequencing platforms still suffer from various issues despite their rapid evolution^103^. They might not produce reads that are 100% aligned with the client requirements due to platform-specific biases^104^, and might generate different types of errors in read data in varying amounts such as low quality bases and PCR errors^105,106^. For an example, some Illumina sequencing platforms have a tendency to produce reads that have lower base quality at the beginning and towards the end of the read^107^. Since most tools in downstream analyses are not capable of detecting these errors during run-time, initial cleaning and filtering of the raw sequence reads is critical to obtain accurate results in analyses.

Several tools have been developed to identify and evaluate the read quality. Such tools can visualize base quality scores, nucleotide distributions, contamination and GC bias. Among them, FASTX-toolkit^77^, which consists of several Linux command line tools, has been widely used. FASTX-toolkit is capable of checking base quality and nucleotide distribution of short-read FASTQ files. However, FASTX-toolkit only supports Illumina reads. Further, FastQC package^76^ can also be used to generate insights about the raw reads. The SortMeRNA^79^ tool can also clean rRNA from data generated from several platforms including Illumina, IonTorrent and PacBio.

However, if the read quality is extremely poor, re-sequencing would be the best option, since filtering alone would not correct such extreme errors. In addition, these errors would further escalate as the data proceeds along the workflow. Nevertheless, quality control will allow the identification of bad samples at early stages, reducing the amount of time spent on analyzing the data at later stages of the workflow. This would result in higher accuracy in the post-processing of data.

While a plethora of assemblers have been developed for assembly processes, it is important to choose an assembler that fits the requirements of the research. For an example, most genome assemblers are not optimized to carry out transcriptome assembly^108^, and reference-based assemblers are not optimized for de novo assembly. Here we used Trinity de novo assembler^80^, since our data were short-read paired end RNA-seq reads of a non-model organism. Trinity is considered to provide high quality assemblies^81^.

Other popular software for de novo assembly of short-read RNA-seq data includes SOAPdenovo-Trans^108^, Oases^109^, and Trans-ABySS^110^ which have been successfully applied in assembling the transcriptomes of various organisms^30,31,111,112^. All of these assemblers use de Bruijn graphs (DBG) to construct the transcriptome assembly. The DBG method is most popularly employed for short-read assembly, and uses a form of K-mer graphs to build the assembly from raw reads^113^.

SOAPdenovo-Trans is derived from SOAPdenovo2 genome assembler^114^, developed as a solution for the algorithmic challenge of assembling very short paired end RNA-seq reads into complete or full-length transcript sequences. Similarly, Oases was extended from Velvet assembler which was originally developed for genome assembly. Trans-AbySS addresses variations in local read densities. Trinity was developed by the Broad Institute in collaboration with the Hebrew University of Jerusalem and is a good general solution for de novo assembly as well as genome-guided transcriptome assembly. It applies three software modules sequentially on large volumes of RNA-seq data, and correct transcript reconstruction is ensured since it was specially programmed to remove ambiguous and erroneous reads. Further, Trinity has assembly options for both single-end and paired-end reads.

Trinity also provides the option of normalizing the reads before assembling. Normalizing is usually required for large datasets with deep sequencing, as redundant data could be present^115^. This would efficiently reduce the computing requirements for de novo assembly including time and memory requirements while retaining the sequencing coverage^116^. Nevertheless, not all datasets require normalization. If enough computing power is available, smaller datasets with lower coverage can be assembled without normalizing, as normalization would discard only the reads that are above a high coverage threshold^117^. Therefore, it is important to correctly identify the transcriptome complexity as well as read properties before normalizing data. We recommend performing assembly both with and without normalization, and evaluate the assembly quality to identify the best course of action. While stand-alone normalization tools such as the khmer software package ^118^ are available, Trinity conveniently provides in silico normalization^82^ in the pipeline itself. Trinity also bundles Trimmomatic with it, enabling the user to trim and correct low quality reads and adapter sequences. Trinity normalizes, trims, and assembles consecutively with one command with necessary input parameters.

By a majority of the results, the *S. album* transcriptome assembly can be considered to be of good quality. However, the discrepancy between the metrics generated by various evaluation tools shows that further assessments are needed to validate the assembly. Such discrepancies are not uncommon^119^ and reasons are discussed by several authors^56,99,100^ . The original study from which we obtained data had followed a somewhat different methodology in preparing the sequence reads for the transcriptome assembly, but had also used Trinity for assembly^70^. The mean length of the transcripts of their assembly was 864 bp. The transcriptome we assembled had transcripts with a mean length of 814.88 bp, which indicates the assemblies were similar in quality. One of the options to improve the overall quality would be to re-sequence with different specifications.

Nevertheless, the expected quality depends on the downstream applications. For example, if the assembly is used for read mapping and differential expression analysis, the initial set of transcripts might be sufficient^120^. For genetic diversity studies and evolutionary assessments^121^, high quality assemblies might be required. However, an assembly with extremely poor quality should not be used for any downstream analyses as it might not represent the genomic or transcriptomic information about the organism accurately^122^. Simple errors can accumulate into significant mis-assemblies^123^. These might result in inaccurate reconstructions of the genome/transcriptome, leading to false results and conclusions.

SSR primer design is one of the downstream applications of assembled transcriptomes. Here we considered several oil biosynthesis genes of *S. album* with the objective of developing gene specific markers for future breeding efforts. While all the primers tested resulted clear PCR products, the product sizes were greater than the expected sizes for KT160233, KT160234 and KT160239. The larger size may attribute to genetic differences in number of repeats in the SSR motif between different accessions. While the RNA-seq data was from an Australian *S. album* accession, validation was done with a Sri Lankan accession. Further, multiple alleles of KT160236 present in the selected accession did not appear in the bioinformatics analysis. Such kind of polymorphism is common in SSR motifs^98^.

Interestingly, the genetic polymorphism observed is correlated with the related chemical constituents of the genotype (data not shown), suggesting their usefulness in breeding programs. While many published work are available on SSR primer design flows^124,125^, only a few had combined bioinformatics with wet lab validations^126–128^. Current data suggests the necessity of such validations to capture the naturally existing biological variation, very common especially in the cross-pollinating or out-crossing species.

## Conclusions

In this paper, we provided a methodology to be followed in assembling a transcriptome from raw data, as well as evaluate the accuracy of the assembly. In order to simplify the process and make it more comprehensible, we used freely available software and tools for the entire workflow. This allows the researcher to experiment and understand the flow of work without external challenges. While the presented methodology discusses the most popular tools used at each stage, it is recommended that the necessary tools are chosen according to the characteristics of the data as well as the end goal of the transcriptome assembly. Furthermore, we utilized the assembled transcriptome for one important application – identification of gene-specific SSR markers – for *S. album* breeding programs. All the designed markers amplified successfully, validating the designed workflow.

To best of our knowledge, this is the first validated attempt of a bioinformatics workflow for de novo transcriptome assembly followed by SSR primer design using freely available software. Most importantly, the bits and pieces of the process are connected in a user-friendly manner, facilitating efforts of biologists. Most of the available pipelines for transcriptome assembly are completely automated, and the work packages are bundled together. Other than a few environmental configurations, the user is not asked to manually examine or handle intermediate outputs during the assembly process in these pipelines. Rather, they are provided with the means to supply raw sequence data as input to the pipeline, and receive a complete or draft transcriptome assembly as the final output. A drawback of this fully-automated approach is that novel biologists may find it too ambiguous as to what happens during the assembly process. As a solution, our workflow is separated into individual modules that are executed separately. This allows the user the flexibility to observe and handle intermediate files, providing a greater depth of understanding as to what is happening with the data at each stage of the process. For beginner biologists, this would be very helpful in understanding the fundamentals of RNA-seq data and transcriptome assembly.

Also, having individual scripts for each of the stages means that the user could easily use different individual scripts simultaneously on different sets of data without affecting the outcome. For an example, the user can pre-process the dataset A using the relevant script for quality control, while at the same time running the transcriptome assembly script on dataset B that had been already pre-processed, even as they are designing primers for dataset C. This would allow users to work efficiently, while working on multiple analyses simultaneously. Having a bundled, automated end-to-end pipeline would prevent the user from using it on multiple datasets which are at different processing stages at once. Another advantage of the separated components is that the user can easily branch out or extend the workflow into other experiments by integrating new user-defined or already available tools. If analysis priorities were to change, it should be relatively easy to modify and re-direct the workflow. This would prove very useful in building an in-house RNA-seq assembly and analysis system for research teams and labs at no cost. In addition, since no complex set-up of the environment is expected of the user other than installing the necessary individual programs, anyone without great knowledge or background in computer science could easily use the scripts to analyze their data.

Therefore, it is evident that our workflow for de novo transcriptome assembly and SSR primer design is simple, comprehensive in dealing with necessary stages required to assemble the transcriptome and design SSR primers, yet complete in providing a workflow starting from raw RNA-seq data to analysis. Considering that it extends to primer design, it is a unique workflow among the cohort of transcriptome assembly pipelines. It is favorable for small institutions and research teams, as a solution for their RNA-seq analysis needs under very low budgets but greater research objectives.

## Supplementary information

Supplementary Information

## Data Availability

The RNA_seq dataset analysed during the current study is publicly available in the NCBI repository, deposited by a previously published paper (BioProject PRJNA297453), https://trace.ncbi.nlm.nih.gov/Traces/study/?acc=SRP064355. The gene data are also publicly available at accessions KC842188.1, KT160233.1, KT160234.1, KT160235.1 , KT160236.1, KT160237.1, KT160238.1, KT160239.1, NC_000932.1 (54,958.0.56397), and NC_003070.9 (28,451,138.0.28453820, complement) in the NCBI repository under previous original submissions.

## Abbreviations

CTAB: Cetyl trimethyl ammonium bromide
DBG: De Bruijn graph
GB: Giga bytes
Gb: Giga bases
NGS: Next generation sequencing
PCR: Polymerase chain reaction
SSR: Simple sequence repeat
