# Supplementary material for: Raw transcriptomics data to gene specific SSRs: a validated free bioinformatics workflow for biologists
Source: Sci Rep. 2020 Oct 26;10:18236. doi: 10.1038/s41598-020-75270-8 (PMC7588437; doi:10.1038/s41598-020-75270-8)
Supplement: Supplementary file 1 — Supplementary Information [file 41598_2020_75270_MOESM1_ESM.pdf]

Raw Transcriptomics Data to Gene Specific SSRs: A Validated Free Bioinformatics Workflow for Biologists

D. N. U. Naranpanawa[1,2], C. H. W. M. R. B. Chandrasekara[1], P. C. G. Bandaranayake[1], A. U. Bandaranayake[3*]

Supplementary file 01

```
#!/bin/bash -ve

#Please save this file as Supplementary file 01.sh for execution

#Execution command
  #sudo nohup bash Supplementary file 07.sh &

#Disclaimer: The download links for the software may be outdated, broken, etc. Please edit as necessary when installing.

#Requirements
#64-bit Linux OS
#Python3
#Java JDK

#find path to your current directory
dir="$PWD"

#install SRAtoolkit
wget --output-document sratoolkit.tar.gz http://ftp-trace.ncbi.nlm.nih.gov/sra/sdk/current/sratoolkit.current-ubuntu64.tar.gz
tar -vxzf sratoolkit.tar.gz
rm -rf sratoolkit.tar.gz
mv sratoolkit* sratoolkit
echo export PATH='$PATH':$dir/sratoolkit/bin >> ~/.bashrc
echo export SRA_TOOLKIT_HOME=$dir/sratoolkit >> ~/.bashrc

#install FastQC
wget https://www.bioinformatics.babraham.ac.uk/projects/fastqc/fastqc_v0.11.9.zip
unzip fastqc_v0.11.9.zip
rm -rf fastqc_v0.11.9.zip
mv fastqc* fastqc
echo export FASTQC_HOME=$dir/fastqc >> ~/.bashrc

#install FASTX-toolkit
wget https://github.com/agordon/fastx_toolkit/releases/download/0.0.14/fastx_toolkit-0.0.14.tar.bz2
tar -xjf fastx_toolkit-0.0.14.tar.bz2
mv fastx_toolkit* fastx_toolkit
cd fastx_toolkit
./configure
make
make install
echo export FASTX_HOME=$dir/fastx_toolkit >> ~/.bashrc

#install Trimmomatic
wget http://www.usadellab.org/cms/uploads/supplementary/Trimmomatic/Trimmomatic-0.39.zip
unzip Trimmomatic-0.39.zip
rm -rf Trimmomatic-0.39.zip
mv Trimmomatic* Trimmomatic
echo export PATH='$PATH':$dir/Trimmomatic >> ~/.bashrc
echo export TRIMMOMATIC_HOME=$dir/Trimmomatic >> ~/.bashrc

#install SortMeRNA
```

```
wget --output-document sortmerna.tar.gz https://github.com/biocore/sortmerna/archive/2.1.tar.gz
tar -vxzf sortmerna.tar.gz
rm -rf sortmerna.tar.gz
mv sortmerna* sortmerna
cd sortmerna
bash ./build.sh
sudo make install
echo export SORTMERNA_HOME=$dir/sortmerna >> ~/.bashrc

#installing Trinity
wget --output-document trinity.tar.gz
https://github.com/trinityrnaseq/trinityrnaseq/releases/download/v2.11.0/trinityrnaseq-v2.11.0.FULL.tar.gz
tar -vxzf trinity.tar.gz
rm -rf trinity.tar.gz
mv trinityrnaseq* trinityrnaseq
cd trinityrnaseq
make
make plugins
make install
echo export TRINITY_HOME=/usr/local/bin >> ~/.bashrc

#install Bowtie2
wget --output-document bowtie2.zip https://sourceforge.net/projects/bowtie-bio/files/bowtie2/2.4.1/bowtie2-2.4.1-
linux-x86_64.zip/download
unzip bowtie2.zip
rm -rf bowtie2.zip
mv bowtie2* bowtie2
echo export PATH='$PATH':$dir/bowtie2/bowtie2 >> ~/.bashrc
echo export PATH='$PATH':$dir/bowtie2/bowtie2-build >> ~/.bashrc
echo export PATH='$PATH':$dir/bowtie2/bowtie2-build-l >> ~/.bashrc
echo export PATH='$PATH':$dir/bowtie2/bowtie2-build-s >> ~/.bashrc
echo export BOWTIE_HOME=$dir/bowtie2 >> ~/.bashrc

#install prerequisites for BUSCO
#sudo apt-get install python3

#install ncbi-blast
wget --output-document ncbi-blast.tar.gz  https://ftp.ncbi.nlm.nih.gov/blast/executables/blast+/LATEST/ncbi-blast-
2.10.1+-x64-linux.tar.gz
tar -vxzf ncbi-blast.tar.gz
rm -rf ncbi-blast.tar.gz
mv ncbi-blast* ncbi-blast
echo export PATH='$PATH':$dir/ncbi-blast >> ~/.bashrc
echo export PATH='$PATH':$dir/ncbi-blast/bin/blastn >> ~/.bashrc
echo export PATH='$PATH':$dir/ncbi-blast/bin/makeblastdb >> ~/.bashrc

#install hmmer
sudo apt-get install hmmer

#install augustus
wget http://bioinf.uni-greifswald.de/augustus/binaries/augustus.current.tar.gz
tar -xzf augustus.current.tar.gz
rm -rf augustus.current.tar.gz
mv augustus* augustus
echo export PATH='$PATH':$dir/augustus/bin >> ~/.bashrc
echo export PATH='$PATH':$dir/augustus/scripts >> ~/.bashrc
echo export AUGUSTUS_CONFIG_PATH=$dir/augustus/config >> ~/.bashrc

#install BUSCO
wget https://gitlab.com/ezlab/busco/-/archive/master/busco-master.zip
```

```
unzip busco-master.zip
rm -rf busco-master.zip
cd busco-master
sudo python3 setup.py install
echo export BUSCO_CONFIG_FILE=$dir/busco-master/config/config.ini >> ~/.bashrc
echo export BUSCO_HOME=$dir/busco-master >> ~/.bashrc

#install Transrate
wget --output-document transrate.tar.gz https://bintray.com/artifact/download/blahah/generic/transrate-1.0.3-linux-x86_64.tar.gz
tar -vzxf transrate.tar.gz
rm -rf transrate.tar.gz
mv transrate* transrate
echo export PATH='$PATH':$dir/transrate >> ~/.bashrc
echo export TRANSRATE_HOME=$dir/transrate >> ~/.bashrc

source ~/.bashrc
```

Supplementary file 02

```
SRR2541771
SRR2541772
SRR2541773
SRR2541774
SRR2541775
SRR2541776
SRR2541777
SRR2541778
SRR2541779
SRR2541780
SRR2541781
SRR2541792
SRR2541798
SRR2541801
SRR2541805
SRR2541808
SRR2541809
SRR2541810
SRR2541811
SRR2541812
SRR2541813
```

Supplementary file 03

```
#!/bin/bash -ve

#Please save this file as Supplementary file 03.sh for execution

#Execution command
  #sudo nohup bash Supplementary file 03.sh &

#Download data

#Intended software - sratoolkit from NCBI

#To download data from NCBI
	#find out your preferred study from Google Scholar or NCBI itself.
  #Go to the SRA repository - https://trace.ncbi.nlm.nih.gov/Traces/sra/
  #Go to Browse --> Studies --> Enter the BioProject number of the chosen study --> Go
	#In the study page click on Runs --> and download the available 'Accession List' which will be downloaded as SRR_Acc_List.txt
```

```
  #You can replace the Supplementary file 02.txt with SRR_Acc_List.txt


filename="Supplementary file 02.txt"
while read -r line
do
    name="$line"
    $SRA_TOOLKIT_HOME/bin/prefetch "$name"

done < "$filename"

#Converting the cached data files into .fastq format

while read -r line
do
    name="$line"
    $SRA_TOOLKIT_HOME/bin/fastq-dump --defline-seq '@$sn[_$rn]/$ri' --split-files "$name"
done < "$filename"
```

Supplementary file 04

```
#!/bin/bash -ve

#Please save this file as Supplementary file 04.sh for execution

#Execution command
  #sudo nohup bash Supplementary file 04.sh &

#Quality check

#This script automates the quality check process of your raw reads

#Intended software - FastQC and FASTX-toolkit

#find path to your current directory
dir="$PWD"

#Specify your file with accession names
filename="Supplementary file 02.txt"

#create a list with your accession numbers
declare -a LIST

# Load file into list.
let i=0
while IFS=$'\n' read -r line_data; do
    LIST[i]="${line_data}"
    ((++i))
done < "$filename"

#Run FASTQC on all files
#A separate output directory will be created for each file for ease of observation
for VALUE in "${LIST[@]}"
do
            mkdir ${VALUE}_1_fastqc
            mkdir ${VALUE}_2_fastqc
            fastqc --outdir="$dir"/${VALUE}_1_fastqc ${VALUE}_1.fastq
            fastqc --outdir="$dir"/${VALUE}_2_fastqc ${VALUE}_2.fastq
done
```

```
#Run FASTX-toolkit on all files
#Change quality filters as necessary

for VALUE in "${LIST[@]}"
do
	fastq_quality_filter -v -q 20 -p 75 -i ${VALUE}_1.fastq -o ${VALUE}_1_filtered.fastq >
${VALUE}_1_FASTX.txt
	fastq_quality_filter -v -q 20 -p 75 -i ${VALUE}_2.fastq -o ${VALUE}_2_filtered.fastq >
${VALUE}_2_FASTX.txt
done
```

Supplementary file 05

```
#!/bin/bash -ve

#Please save this file as Supplementary file 06.sh for execution

#Execution command
  #sudo nohup bash Supplementary file 06.sh &

#Trim adapters with Trimmomatic

#Intended software - Trimmomatic

#find path to your current directory
dir="$PWD"

#Specify your file with accesison names
filename="Supplementary file 02.txt"

#create a list with your accession numbers
declare -a LIST

# Load file into list.
let i=0
while IFS=$'\n' read -r line_data; do
    LIST[i]="${line_data}"
    ((++i))
done < "$filename"

for VALUE in "${LIST[@]}"
do
  java -Xmx1G -jar $TRIMMOMATIC_HOME/trimmomatic-0.38.jar PE -phred33 -threads 16 -trimlog
${VALUE}logfile \
  "$dir"/${VALUE}_non_rRNA_1.fastq "$dir"/${VALUE}_non_rRNA_2.fastq \
  ${VALUE}_left_P_qtrim.fq ${VALUE}_left_U_qtrim.fq ${VALUE}_right_P_qtrim.fq
${VALUE}_right_U_qtrim.fq \
  ILLUMINACLIP:$TRIMMOMATIC_HOME/adapters/TruSeq3-PE.fa:2:30:10 SLIDINGWINDOW:5:20
LEADING:5 TRAILING:5 MINLEN:50
done
```

Supplementary file 06

```
#!/bin/bash -ve

#Please save this file as Supplementary file 05.sh for execution

#Execution command
  #sudo nohup bash Supplementary file 05.sh &
```

```
#Removing ribosomal RNA (rRNA)

#Intended software - Sortmerna

#find path to your current directory
dir="$PWD"

$SORTMERNA_HOME/indexdb_rna --ref \
$SORTMERNA_HOME/rRNA_databases/silva-bac-16s-id90.fasta,$SORTMERNA_HOME/index/silva-bac-16s-db:\
$SORTMERNA_HOME/rRNA_databases/silva-bac-23s-id98.fasta,$SORTMERNA_HOME/index/silva-bac-23s-db:\
$SORTMERNA_HOME/rRNA_databases/silva-arc-16s-id95.fasta,$SORTMERNA_HOME/index/silva-arc-16s-db:\
$SORTMERNA_HOME/rRNA_databases/silva-arc-23s-id98.fasta,$SORTMERNA_HOME/index/silva-arc-23s-db:\
$SORTMERNA_HOME/rRNA_databases/silva-euk-18s-id95.fasta,$SORTMERNA_HOME/index/silva-euk-18s-db:\
$SORTMERNA_HOME/rRNA_databases/silva-euk-28s-id98.fasta,$SORTMERNA_HOME/index/silva-euk-28s:\
$SORTMERNA_HOME/rRNA_databases/rfam-5s-database-id98.fasta,$SORTMERNA_HOME/index/rfam-5s-db:\
$SORTMERNA_HOME/rRNA_databases/rfam-5.8s-database-id98.fasta,$SORTMERNA_HOME/index/rfam-5.8s-
db


#create a list with your accession numbers
filename="Supplementary file 02.txt"
declare -a LIST
let i=0
while IFS=$'\n' read -r line_data; do
    LIST[i]="${line_data}"
    ((++i))
done < "$filename"

for VALUE in "${LIST[@]}"
do
  $SORTMERNA_HOME/scripts/merge-paired-reads.sh ${VALUE}_1.fastq ${VALUE}_2.fastq
${VALUE}_interleaved.fastq
$SORTMERNA_HOME/sortmerna --ref \
$SORTMERNA_HOME/rRNA_databases/silva-bac-16s-id90.fasta,$SORTMERNA_HOME/index/silva-bac-16s-db:\
$SORTMERNA_HOME/rRNA_databases/silva-bac-23s-id98.fasta,$SORTMERNA_HOME/index/silva-bac-23s-db:\
$SORTMERNA_HOME/rRNA_databases/silva-arc-16s-id95.fasta,$SORTMERNA_HOME/index/silva-arc-16s-db:\
$SORTMERNA_HOME/rRNA_databases/silva-arc-23s-id98.fasta,$SORTMERNA_HOME/index/silva-arc-23s-db:\
$SORTMERNA_HOME/rRNA_databases/silva-euk-18s-id95.fasta,$SORTMERNA_HOME/index/silva-euk-18s-db:\
$SORTMERNA_HOME/rRNA_databases/silva-euk-28s-id98.fasta,$SORTMERNA_HOME/index/silva-euk-28s:\
$SORTMERNA_HOME/rRNA_databases/rfam-5s-database-id98.fasta,$SORTMERNA_HOME/index/rfam-5s-db:\
$SORTMERNA_HOME/rRNA_databases/rfam-5.8s-database-id98.fasta,$SORTMERNA_HOME/index/rfam-5.8s-
db \
--reads "$dir"/${VALUE}_interleaved.fastq --num_alignments 1 \
--fastx --aligned ${VALUE}_rRNA --other ${VALUE}_non_rRNA --log -a 8 -m 64000 --paired_in -v
done

#unmerge your rRNA-free files
for VALUE in "${LIST[@]}"
do
$SORTMERNA_HOME/scripts/unmerge-paired-reads.sh ${VALUE}_non_rRNA.fastq
${VALUE}_non_rRNA_1.fastq ${VALUE}_non_rRNA_2.fastq
done
```

Supplementary file 07

```
#!/bin/bash -ve

#Please save this file as Supplementary file 07.sh for execution

#Execution command
```

```
  #sudo nohup bash Supplementary file 07.sh &

#Normalize data

#Intended software - Trinity de novo assembler

#find path to your current directory
dir="$PWD"

#Specify your file with accesison names
filename="Supplementary file 02.txt"

#create a list with your accession numbers
declare -a LIST

# Load file into list.
let i=0
while IFS=$'\n' read -r line_data; do
    LIST[i]="${line_data}"
    ((++i))
done < "$filename"

for VALUE in "${LIST[@]}"
do
  perl $TRINITY_HOME/util/insilico_read_normalization.pl --seqType fq --JM 100G --max_cov 50 \
  --left "$dir"/${VALUE}_left_P_qtrim.fq --right "$dir"/${VALUE}_right_P_qtrim.fq \
  --pairs_together --PARALLEL_STATS --CPU 8 --output "$dir"/
done
```

Supplementary file 08

```
#!/bin/bash -ve

#Please save this file as Supplementary file 08.sh for execution

#Execution command
  #sudo nohup bash Supplementary file 08.sh &

#De novo assemble RNA-seq data

#Intended software - Trinity de novo assembler

#find path to your current directory
dir="$PWD"

#Concatenate normalized right reads and normalized left reads separately

cat *_left_P_qtrim.fq.normalized_*.fq > left.fq
cat *_right_P_qtrim.fq.normalized_*.fq > right.fq

#Assemble

$TRINITY_HOME/Trinity --seqType fq --SS_lib_type RF \
--left "$dir"/left.fq \
--right "$dir"/right.fq \
--CPU 8 --max_memory 100G --output "$dir"/trinity_out
```

Supplementary file 09

```
#!/bin/bash -ve

#Please save this file as Supplementary file 09.sh for execution

#Execution command
  #sudo nohup bash Supplementary file 09.sh &

#Quality assessment of transcriptome assembly

#Intended software - Trinity de novo assembler, Bowtie2, BUSCO, Transrate

dir="$PWD"

#1. Considering basic statistics
	#Intended software - Trinity de novo assembler

perl $TRINITY_HOME/util/misc/get_longest_isoform_seq_per_trinity_gene.pl Trinity.fasta > Trinity.longest.fasta
perl $TRINITY_HOME/util/TrinityStats.pl Trinity.fasta > basic_stats.txt

#2. Considering Bowtie statistics
	#Intended software - bowtie2

bowtie2-build Trinity.fasta Trinity.fasta

#left.fastq is the forward read file and right.fastq is the reverse read file of original raw sequence data.

bowtie2 --local --no-unal -x Trinity.fasta -q -1 "$dir"/left.fastq -2 "$dir"/right.fastq \
        | samtools view -Sb - | samtools sort -no - - > bowtie2.nameSorted.bam

#3. Considering BUSCO startistics
	#Intended software - BUSCO

#eukaryota_odb9 is the reference BUSCO database. It should be downloaded into your working directory.

python $BUSCO_HOME/scripts/run_BUSCO.py -i /"$dir"/trinity_out/Trinity.fasta \
-o buscoassessment \
-l "dir"/eukaryota_odb9/ -m tran

#4. Considering transrate statistics
	#Intended software - TransRate

#left.fastq is the forward read file and right.fastq is the reverse read file of original raw sequence data.

$TRANSRATE_HOME/transrate --assembly "$dir"/trinity_out/Trinity.fasta \
  --left "$dir"/left.fastq \
  --right "$dir"/right.fastq \
  --output="$dir"/transrate_results/

  #After execution has finished, all assembly quality statistics will be present in nohup.out
```

Supplementary file 10

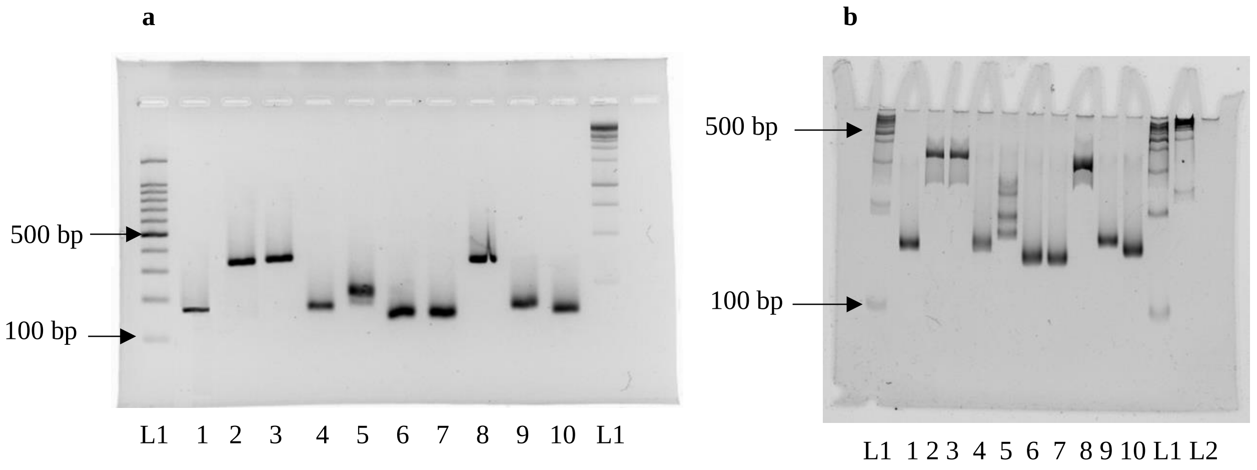

Full length gel image for Figure 4: Polymerase chain reaction amplification of Simple Sequence Repeat markers and two housekeeping genes of *S. album*. (a): Agarose gel electrophoresis (b): Polyacrylamide gel electrophoresis. 1:KC842188.1, 2:KT160233.1, 3:KT160234.1, 4:KT160235.1, 5:KT160236.1, 6: KT160237.1, 7:KT160238.1, 8:KT160239.1, 9:rBcL, 10:TUB1, L1:100 bp molecular weight marker (promega G2101), L2: 1 kb molecular weight marker (promega G5711)

Supplementary file 11

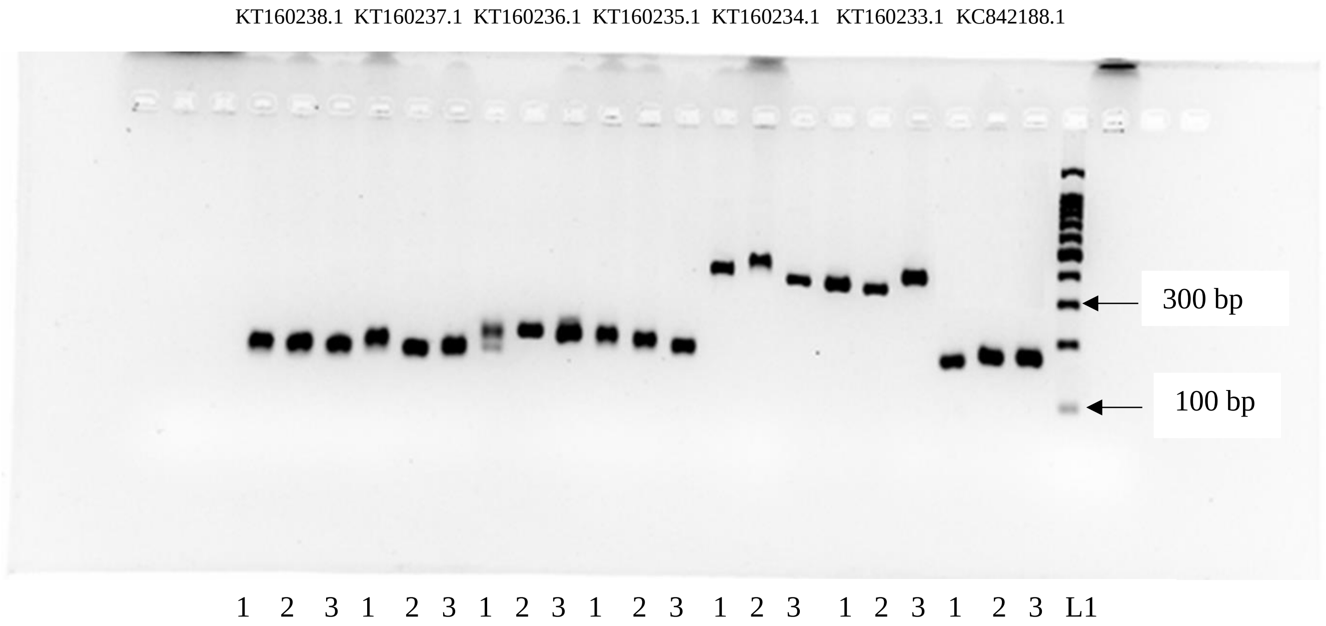

Full length gel image for Figure 5: Agarose gel electrophoresis of Simple Sequence Repeats amplified products of three Sri Lankan *S. album* accessions. L:100 bp molecular weight marker (promega G2101), 1-3: *S. album* accessions
